# Malic enzyme-based system for transhydrogenation between nicotinamide cofactors

**DOI:** 10.1016/j.synbio.2025.08.009

**Published:** 2025-08-21

**Authors:** Haizhao Xue, Yinghan Hu, Aabid Manzoor Shah, Xueying Wang, Xiaojia Guo, Yanzhe Huang, Zongbao K. Zhao

**Affiliations:** a Dalian Institute of Chemical Physics, Chinese Academy of Sciences, Dalian, 116023, China; b University of Chinese Academy of Sciences, Beijing, 100049, China; c MOE Key Laboratory of Bio-Intelligent Manufacturing, School of Bioengineering, Dalian University of Technology, Dalian, 116024, China

**Keywords:** Malic enzyme, Redox cofactor, Nicotinamide cytosine dinucleotide, Transhydrogenation, Energy metabolism

## Abstract

The nicotinamide cofactors including nicotinamide adenine dinucleotide (NAD) and its phosphate (NADP) play important roles in facilitating redox reactions for energy metabolism and biosynthesis. To expand the cofactor menu, a non-natural cofactor nicotinamide cytosine dinucleotide (NCD) has been introduced recently. The reduced forms of these cofactors carry reducing equivalents that are essential for cellular metabolism. However, there is a long standing challenge to rationally transfer reducing equivalent from one cofactor to another, albeit such process is highly demanding in metabolic engineering. This study develops a new approach based on malic enzyme (ME)-mediated transhydrogenation to enable reducing equivalents exchange among different cofactors. We used wild-type ME, MaeB and an engineered ME∗ that favors NAD, NADP and NCD, respectively, to demonstrate such conversions. When an *in vitro* system initiated with equal amount of NADH and NCD in the presence of ME, ME∗ and excess amount of pyruvate was held for 2 h, up to 65 % NADH was consumed and 57 % NCDH was generated. When implemented into NCD self-sufficient *Escherichia coli* cells, the system directed reducing equivalents toward NCDH-linked formation of lactate. Overall, this work offers an effective strategy to regulate intracellular reducing equivalents that may serve as a novel tool for metabolic engineering and synthetic biology.

## Graphical abstract

**Figure:**
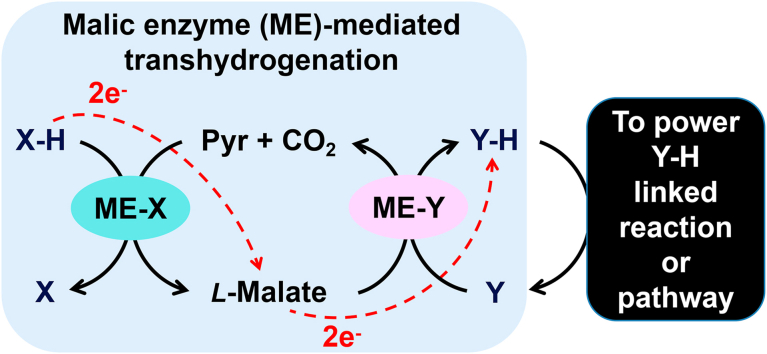

## 1 Introduction

**Fig. 1:**
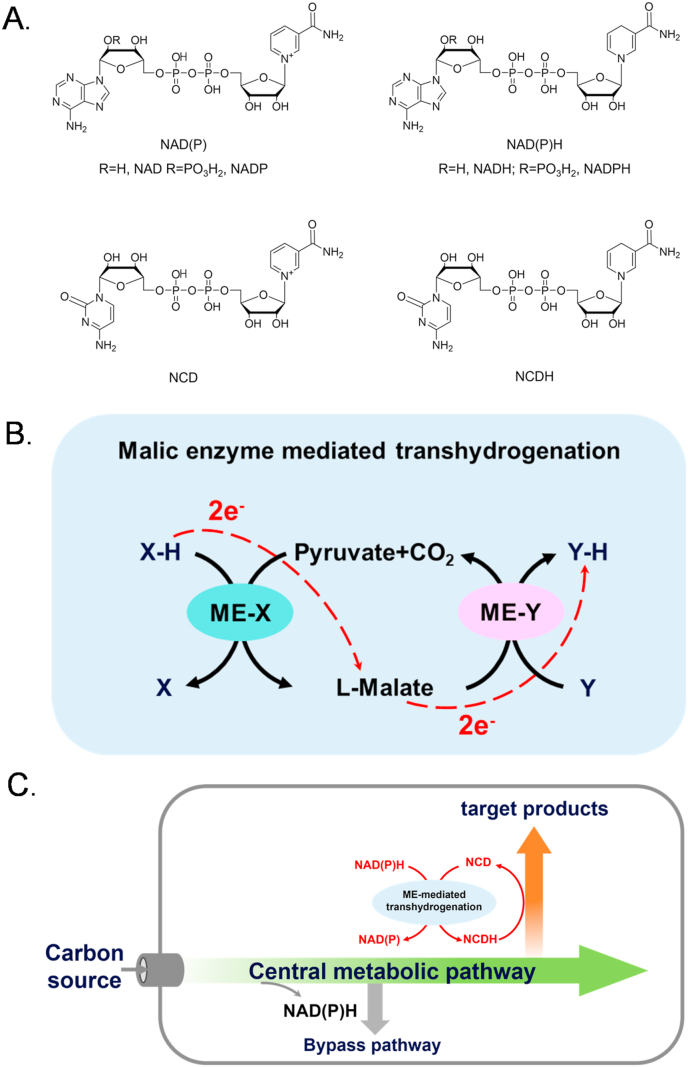
ME-mediated transhydrogenation for regulation of redox cofactors and pathways. A. Chemical structures of redox cofactors. B. ME-mediated transhydrogenation and its electron transferring path. X and Y represent two types of redox cofactors, X–H and Y–H represent the reduced forms of X and Y, and ME-X and ME-Y refer to malic enzymes dependent on X and Y, respectively. C. ME-mediated transhydrogenation to direct reducing equivalents from natural cofactors to NCDH for the biosynthesis of target products.

Nicotinamide cofactors including nicotinamide adenine dinucleotide (NAD), NAD 2′-phosphate (NADP) and their corresponding reduced forms, NADH and NADPH, are integral to cellular metabolism in facilitating redox reactions for energy production and biosynthesis [1]. These cofactors are not only essential for enzyme catalysis but also play a critical role in regulating cellular processes such as growth, differentiation, and stress responses [2]. NAD primarily supports catabolic reactions, where it facilitates the oxidation of substrates to generate energy, while NADP is predominantly involved in anabolic processes for the synthesis of key biomolecules such as fatty acids, amino acids and nucleotides [3]. To expand the cofactor menu, non-natural cofactors that mimic the function of natural cofactors but with altered structures have been explored recently [4,5]. These non-natural cofactors are typically more stable and less expensive. Meanwhile, non-natural cofactors may be used to allocate reducing equivalents, modulate reaction thermodynamics, and ultimately improve product yields, presenting substantial advantages in synthetic biology and biomanufacturing [[6], [7], [8]]. In particular, nicotinamide cytosine dinucleotide (NCD, Fig. 1A) has been found with good biocompatibility, and several enzymes have been engineered to favor NCD, including malic enzyme [9], lactate dehydrogenase [10], formate dehydrogenase [11], formaldehyde dehydrogenase [12], and P450 BM3 [13].

The NADH/NAD and NADPH/NADP ratios are sensitive biochemical indexes for cellular metabolism and the cell redox state [14]. During the catabolic processes, NAD acts as an electron acceptor, stripping electrons from the metabolic substrates to form NADH, which then transfer electrons to oxygen or other electron acceptors, thus cells typically maintain a lower NADH/NAD ratio. On the other hand, anabolic processes require a higher NADPH/NADP ratio, as NADPH serves as a reducing agent in biosynthetic pathways, enabling the construction of complex molecules from simpler precursors [15]. Therefore, it is important to allocate the reducing equivalents for optimal flux of metabolites toward a desired pathway [16]. Furthermore, the non-specific delivery of reducing equivalents may derail metabolic processes, resulting in excess by-products. The dynamic equilibrium of NADH/NAD and NADPH/NADP ratios can be achieved by modulating the activities of key enzymes, utilizing distinct cofactors for substrate-level phosphorylation, transferring hydrogen anion between NAD and NADP, and other mechanisms.

Despite tremendous efforts in developing tools to regulate NAD(P)H/NAD(P) ratios, challenges remain in effectively catalyze the interconversion between NADH and NADPH. So far, transhydrogenase [17,18], glyceraldehyde-3-phosphate dehydrogenase [19], and glutamate dehydrogenase [20,21] have been explored to regulate cellular NAD(P)H levels in metabolic engineering. Moreover, with the introduction of non-natural cofactors, it appears interesting to establish the transhydrogenation process between natural and non-natural cofactors. For instance, when rapid or excessive carbon source uptake triggers overflow metabolism, where reducing equivalents and carbon flux are sequestered into undesirable byproducts. This metabolic imbalance may be strategically resolved by redirection of reducing equivalents into target pathways mediated by non-natural cofactors. While such processes are highly demanding in metabolic engineering and synthetic biology, to date, transhydrogenases have never been characterized or engineered for recognition of non-natural redox cofactors [[22], [23], [24]]. In the absence of known cellular enzymatic mechanisms bridging natural and non-natural cofactors for the transfer of reducing equivalents, alternative strategies are essential to overcome this limitation and enable orthogonal provision of reductive equivalents.

This study aims to develop a new transhydrogenation tool to facilitate reducing equivalents transfer between different redox cofactor pairs, with a particular focus on transhydrogenation between natural and non-natural cofactors. We employed malic enzymes with distinct cofactor specificities, including NAD-dependent ME [25], NCD-dependent ME∗ [9] (ME mutant with L310R/Q401C), and NADP-dependent MaeB [26], as functional components. Pyruvate, CO_2_ and l-malate serve as intermediate substrates for the transhydrogenation process, where l-malate acts as a carrier molecule for hydride in the transfer of reducing equivalents between distinct cofactors (Fig. 1B). Because pyruvate is a central metabolite at relatively high concentrations [27], for example, over 3.0 mM in *Escherichia coli* cells and 8.0 mM in *Saccharomyces cerevisiae* cells, it can provide a driving force for the oxidative half of the process and receive electrons from the reduced cofactor X–H.

to form l-malate. In the reductive half of the process, l-malate delivers hydride to the oxidized cofactor Y to form YH. Thus, the net result of this system may be used to direct the reducing equivalents towards YH-linked reactions or pathways. It is noteworthy that this reduction equivalents transfer can occur between natural cofactors and non-natural cofactors. In *in vitro* experiments, our results demonstrated that the ME-mediated transhydrogenation promoted efficient hydrogen transfer from one cofactor to another one. By implementing this system into NCD self-sufficient *E. coli* cells [28], we found that NCDH-linked formation of lactate was enhanced likely because reducing equivalents carried by natural cofactors were transferred to form NCDH (Fig. 1C). Overall, this work offers an effective strategy to regulate intracellular reducing equivalents that may serve as a novel tool in the fields of metabolic engineering and synthetic biology.

## 2 Materials and methods

### 2.1 Strains and plasmids

The strains and plasmids used in this study are listed in Supplementary Material Table S1. *E*. *coli* BL21(DE3) was used for protein production, and *E. coli* BW25113 (*△ldhA*, *dld::cat*) [28] was used as the host for d-lactate accumulation. All *E. coli* strains were pre-cultured at 37 °C for activation and at 30 °C for protein production.

### 2.2 Reagents and kits

Molecular cloning kits were purchased from Sangon Biotech (Shanghai, China). One step cloning kit was from YEASENBIO (Shanghai, China). Yeast extracts and tryptone were obtained from Oxoid (Basingstoke, UK). PrimeSTAR HS DNA Polymerase and *Dpn*I were sourced from TaKaRa (Dalian, China). The Ni-NTA protein purification resin was obtained from Invitrogen (Carlsbad, USA). NCD and NCDH were synthesized in our laboratory [29]. Alcohol dehydrogenase (ADH II) was obtained from Sigma-Aldrich (Shanghai, China). Coenzyme II NADP(H) Content Assay Kit was from Solarbio (Beijing, China). d-Lactate Assay Kit (K-DATE) and Acetic Acid Assay Kit (K-ACET) were from Megazyme (Bray, Ireland). Amplex Red Glycerol Assay Kit was purchased from Beyotime Biotechnology (Shanghai, China). All other chemicals were sourced from Sigma-Aldrich or Aladdin (Shanghai, China).

### 2.3 Protein production and purification

For ME and ME∗ production, *E. coli* BL21(DE3) cells transformed with the protein production plasmids were cultured in LB medium supplemented with 50 μg/mL kanamycin at 37 °C for 12 h. The culture was then diluted 100-fold into fresh LB medium containing 0.1 mM IPTG and 50 μg/mL kanamycin, and incubated for an additional 48 h at 30 °C. Cells were harvested by centrifugation at 8000×*g*, 4 °C for 5 min, and then frozen at −80 °C. The production of MaeB was described in reference [30]. Protein purification was performed using the Ni-NTA kit. Briefly, cells were resuspended in purification buffer (50 mM sodium phosphate and 500 mM NaCl, pH 8.0), broken by ultrasonication, and centrifuged at 14,000 rpm, 4 °C for 30 min. The supernatant was applied to a Ni-NTA column, washed with purification buffer supplemented with 20 mM imidazole, and the target protein was eluted with purification buffer supplemented with 250 mM imidazole. The purified protein was dialyzed and stored at −80 °C in 50 mM Tris-HCl (pH 7.5) with 20 % glycerol. Protein concentration was determined using a NanoDrop spectrophotometer at 280 nm, and protein purity was analyzed by SDS-PAGE. The molecular weights and molar extinction coefficients of the proteins were calculated using the online tool ExPASy ProtParam. The molar extinction coefficients for ME and ME∗ both were 6.57 × 10^4^ M^−1^ cm^−1^, and for MaeB was 3.08 × 10^4^ M^−1^ cm^−1^. The molecular weights of ME and ME∗ both were 64 kDa, and MaeB was 83 kDa.

### 2.4 Plasmid construction and strain transformation

Vector construction in this study was performed using one step cloning kit. To construct p15A-NCD-ME, the ME [25] gene was amplified from the pET24b-ME using primers 24010502F/24010502R, and pUC-P15A-*Para*-FtNadE-*c*-his-NcdS-2-CtCTPS∗ was linearized by PCR using primers 24010501pF/24010501pR (Table S2). Then, the ME gene was inserted into pUC-P15A-*Para*-FtNadE-*c*-his-NcdS-2-CtCTPS∗ using one step cloning kit. *E. coli* transformation was carried out by electroporation. Molecular verification of the plasmids and strains was performed by sequencing (Sangon Biotech, Shanghai, China).

### 2.5 Activity and kinetic assay

Enzyme activity assays of pure enzymes were conducted in a total volume of 0.1 mL. For malate oxidative decarboxylation, the reaction mixture contained 50 mM HEPES (pH 7.5), 5 mM malate, 2 mM MgCl_2_ and 0.5 mM NAD, NCD, or NADP. The enzyme activity was monitored by measuring the absorbance at 340 nm using a microplate spectrophotometer (Bio-Tek, VT, USA), with continuous absorbance tracking throughout the assay. One unit of pure enzyme activity was defined as the amount of enzyme required to catalyze the production of 1 μmol NADH, NCDH or NADPH per minute under the specified assay conditions. A molar absorption coefficient of 6220 M^−1^ cm^−1^ for NADH, NCDH or NADPH was used in the calculations.

For kinetic assay, the Michaelis-Menten constants *V*_max_ and *K*_m_ of NAD(H), NCD(H), and NADP(H) for the enzymes were obtained from initial rate measurements under conditions in which a coenzyme was varied at different levels. For malic enzymes involved in oxidative decarboxylation, the solution contained 5 mM l-malate and 2 mM MgCl_2_. For malic enzymes involved in reductive carboxylation, the solution included 10 mM pyruvate, 30 mM NaHCO_3_ and 2 mM MgCl_2_. For DLDH∗ involved in pyruvate reduction, initial pyruvate concentration was 10 mM. All reactions were performed in 50 mM HEPES (pH 7.5) at 25 °C, with kinetic data fitted to the Michaelis-Menten equation via nonlinear regression using GraphPad Prism 10.0.2. All assays were performed in triplicate, and error bars show standard deviation.

### 2.6 Transhydrogenation *in vitro*

Transhydrogenation was conducted *in vitro* in a total reaction volume of 0.15 mL. The reaction mixture consisted of 100 mM HEPES (pH 7.5), 2 mM oxidative cofactor, 2 mM reductive cofactor, 0–100 mM pyruvate, 100 mM NaHCO_3_, 2 mM MgCl_2_, and 0.2 U/mL of each malic enzyme. The reactions were carried out at 37 °C. To terminate the reactions, chromatographic-grade methanol was added to the system to a final concentration of 80 %. All reactions were performed in triplicate, and error bars show standard deviation.

### 2.7 Analysis of cofactors by HPLC

The cofactors were analyzed *in vitro* using HPLC, following the method described previously [29]. HPLC detection was carried out with a Huapu High-Performance Liquid Chromatograph (Huapu Scientific Instruments, Beijing). The chromatographic separation was performed on a SUPELCO C18 column.

### 2.8 Biotransformation and analysis

The strains were grown in TB medium supplemented with 50 μg/mL kanamycin, 50 μg/mL carbenicillin, 1 mM IPTG, and 2 mM l-arabinose for 24 h at 30 °C, 200 rpm, with an initial OD_600_ of 0.05. After growth, the cells were collected for analysis of enzyme activities and resting cell catalysis.

For resting cell catalysis, 2 × 10^10^ cells were harvested by centrifugation, washed with 50 mM HEPES (pH 7.5), and resuspended in 1 mL of reaction buffer containing 50 mM HEPES (pH 7.5), 100 mM glucose or glycerol, and 10 mM NaHCO_3_. The reaction was carried out at 37 °C with shaking at 200 rpm. To stop the reaction, 4 vol of the quenching buffer (prepared in a 1:1 *v/v* methanol:acetonitrile mixture) was added, resulting in the sample being preserved in methanol: acetonitrile: water at a final ratio of 2:2:1 (*v/v/v*). The samples were then centrifuged at 10,000×*g* for 2 min at 4 °C, and the supernatant was collected for lactate analysis by d-Lactic Acid Assay Kit (K-DATE) and acetic acid analysis by Acetic Acid Assay Kit (K-ACET) from Megazyme. 3,5-dinitrosalicylic acid (DNS) was used for glucose analysis. The glycerol analysis was carried out by Amplex Red Glycerol Assay Kit from Beyotime Biotechnology.

### 2.9 Resting cells cofactor analysis

Cells during the biotransformation process were collected for cofactor analysis, and intracellular cofactors were extracted as follows [31]. Extraction of intracellular NAD/NCD/NADP: Approximately 2 × 10^10^ resting cells were collected by centrifugation and resuspended in 200 μL of 0.2 M HCl. The mixture was incubated in a 55 °C water bath for 10 min. After incubation, 200 μL of 0.1 M NaOH was added to neutralize the solution. The mixture was then centrifuged, and the supernatant was stored at −80 °C less than a week for further analysis. Extraction of intracellular NADH/NCDH/NADPH: Similarly, 2 × 10^10^ resting cells were collected and resuspended in 200 μL of 0.2 M NaOH, followed by incubation in a 55 °C water bath for 10 min. After neutralization with 200 μL of 0.1 M HCl, the mixture was centrifuged, and the supernatant was stored at −80 °C less than a week for further analysis.

NAD(H) and NCD(H) were assayed by enzymatic cycling assays [28,31], while the difference lies in our utilization of NCD-dependent formate dehydrogenase (FDH∗) [11] for NCD(H) detection, and the schematics was shown in Fig. S1. Briefly, for NAD(H), the assay mixture consisted of 5 U/mL ADH, 100 mM Bicine (pH 8.0), 10 μL of anhydrous ethanol, 0.4 mM thiazolyl blue (MTT), 1 mM phenazine ethosulfate (PES), and 4 mM EDTA (pH 8.0). Absorbance was measured at 570 nm. And for NCD(H), the assay mixture contained 1 mg/mL FDH∗, 50 mM HEPES (pH 7.5), 10 mM formate, 0.4 mM MTT, and 1 mM PES. Absorbance was measured at 570 nm. NADP(H) was assayed by Coenzyme II NADP(H) Content Assay Kit from Solarbio. Concentrations of cofactors within the cells were calculated with data measured according to the methods described in references [32]. All assays were performed in triplicate, and error bars show standard deviation.

### 2.10 Resting cells enzymatic activities determination

To determine the enzymatic activity of crude cell extracts from engineered strains, 2 × 10^10^ cells were harvested and lysed in 0.2 mL of cell lysis buffer containing 10 mM Tris-HCl (pH 8.0), 1 mM MgCl_2_, 0.1 mg/mL DNase I, and 1 mg/mL lysozyme. The lysate was incubated at 37 °C with shaking at 200 rpm for 2 h. After incubation, the mixture was centrifuged, and the supernatant was collected for the activity assays. Enzyme assays of the crude cell extracts were performed in a total reaction volume of 0.1 mL. For the malic enzyme activity, the reaction mixture contained 50 mM HEPES (pH 7.5), 5 mM malate, 0.4 mM MTT, 1 mM PES, 500 μM NAD or NCD, and 10 mM MgCl_2_. For the d-lactate dehydrogenase activity, the reaction mixture included 50 mM HEPES (pH 7.5), 20 mM d-lactate, 0.4 mM MTT, 1 mM PES, and 500 μM NAD or NCD. The enzyme activity was monitored by measuring absorbance at 570 nm using a Universal Microplate Spectrophotometer (Bio-Tek, VT, USA), with continuous absorbance tracking throughout the assay. One unit of crude enzyme activity was defined as the production of 1 μmol formazan per min. All assays were performed in triplicate.

## 3 Results and discussion

### 3.1 ME-mediated transhydrogenation

**Fig. 2:**
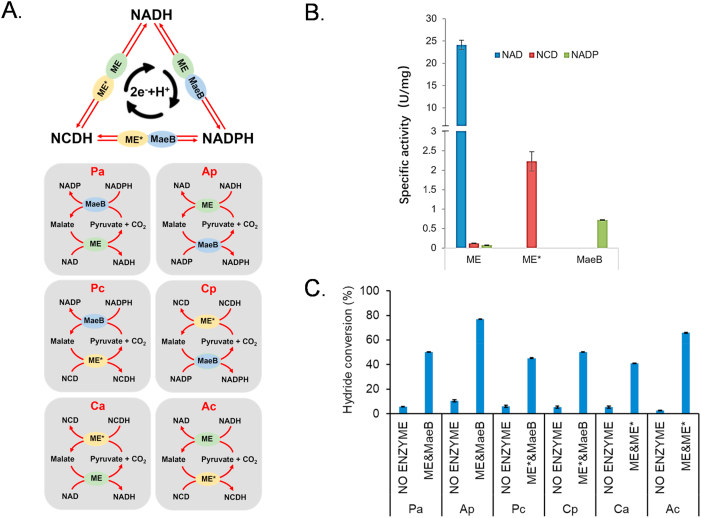
ME-mediated transhydrogenation *in vitro*. A. Diagram of the ME-mediated transhydrogenation. B. Specific enzyme activities of three malic enzymes with different cofactors. C. Conversion of the ME-mediated transhydrogenation *in vitro*. Reactions were done at 37 °C for 60 min, in 100 mM HEPES (pH 7.5) with 2 mM oxidative cofactor, 2 mM reductive cofactor, 50 mM pyruvate, 100 mM NaHCO_3_, 2 mM MgCl_2_ and 0.2 U/mL of each malic enzyme. Pa: NADPH + NAD → NADP + NADH; Ap: NADH + NADP → NAD + NADPH; Pc: NADPH + NCD → NADP + NCDH; Cp: NCDH + NADP → NCD + NADPH; Ca: NCDH + NAD → NCD + NADH; Ac: NADH + NCD → NAD + NCDH. NO ENZYME groups were performed without the addition of any malic enzyme. Experiments were done in triplicate, and error bars indicate standard deviation.

This study designed a hydrogen transfer system mediated by distinct cofactor-dependent malic enzymes, in which the reversible reaction of pyruvate conversion to malate serves as a bridge to facilitate transhydrogenation between different cofactors. On one hand, pyruvate serves as a vital intermediate for the interconversion of diverse substances across all living organisms, with a substantial concentration present within cells. On the other hand, both pyruvate and malate demonstrate exceptional biocompatibility. Additionally, we have obtained well-characterized malic enzymes with different cofactor dependencies as components of our hydrogen transfer system, including NAD-dependent ME [25], NCD-dependent ME∗ [9] (ME mutant with L310R/Q401C), and NADP-dependent MaeB [26], which are all native or derivative proteins of *E. coli*, minimizing the possibility of low expression during recombinant protein production [33]. This approach could facilitate transhydrogenation between NAD, NADP, and the non-natural cofactor NCD (Fig. 1, Fig. 2A).

We expressed and purified three types of malic enzymes (ME, ME∗, and MaeB), each of which demonstrated a clear preference for NAD, NCD, or NADP, respectively, as shown in Fig. S2 and Fig. 2B. Specifically, the activity of ME towards NAD was 24.15 U/mg, the activity of ME∗ towards NCD was 2.23 U/mg, and the activity of MaeB towards NADP was 0.72 U/mg. ME exhibited the highest activity towards NAD and weak activity towards NCD and NADP, whereas neither ME∗ or MaeB activity was detected for the other two cofactors. These enzymes provide the necessary components for the ME-mediated transhydrogenation.

### 3.2 Transhydrogenation *in vitro*

To verify the feasibility of our design, we first conducted catalytic experiments for hydrogen transfer between any two of the cofactors using purified proteins. In these catalytic reactions, half of the transhydrogenation processes was mainly driven by reduced cofactors and pyruvate/CO_2_ from substrates, while the other half depended on oxidizing cofactors and the thermodynamic favorability of malate oxidation and decarboxylation to pyruvate/CO_2_. To ensure the availability of CO_2_ for facilitating the transhydrogenation reaction, we supplemented the reaction system with sodium bicarbonate to increase the accessible CO_2_ concentration. Reactions were conducted at 37 °C for 60 min in a mixture containing 100 mM HEPES, 2 mM oxidative cofactor, 2 mM reductive cofactor, 50 mM pyruvate, 100 mM NaHCO_3_, 2 mM MgCl_2_ and 0.2 U/mL of each malic enzyme, pH 7.5. We employed a short abbreviation to denote transhydrogenation reaction from one cofactor to the other: Pa is used for the transhydrogenation from NADPH to NAD (NADPH + NAD → NADP + NADH), Ap for NADH to NADP (NADH + NADP → NAD + NADPH), Pc for NADPH to NCD (NADPH + NCD → NADP + NCDH), Cp for NCDH to NADP (NCDH + NADP → NCD + NADPH), Ca for NCDH to NAD (NCDH + NAD → NCD + NADH), and Ac for NADH to NCD (NADH + NCD → NAD + NCDH). HPLC analysis demonstrated successful and efficient hydrogen transfer between NAD, NCD, and NADP, achieving hydrogenation conversions ranging from 40 % to 80 %, as shown in Fig. 2C. Specifically, the conversions for Ap (NADH + NADP → NAD + NADPH) and Ac (NADH + NCD → NAD + NCDH) were 76.9 % and 65.9 %, respectively. Notably, the NO ENZYME groups without any malic enzyme only exhibited less than 10 % conversion. This demonstrates the feasibility of employing malic enzymes to mediate hydrogen transfer, thereby enabling controlled utilization of both natural and non-natural cofactors in redox reactions.

**Fig. 3:**
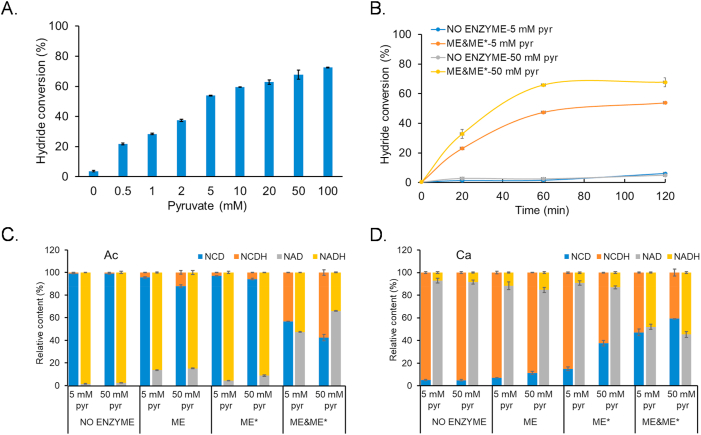
*In vitro* characterization of transhydrogenation between NADH and NCDH. A. Effects of pyruvate concentration on the transhydrogenation for Ac (NADH + NCD → NAD + NCDH). The system was initiated with 100 mM HEPES (pH 7.5), 2 mM NADH, 2 mM NCD, 100 mM NaHCO_3_, 2 mM MgCl_2_, 0.2 U/mL of both ME and ME∗, held at 37 °C for 120 min. B. Time courses of the transhydrogenation processes. Ac (NADH + NCD → NAD + NCDH), 5 or 50 mM pyruvate was used. C. Relative contents of redox cofactors after 60 min, Ac (NADH + NCD → NAD + NCDH), 5 or 50 mM pyruvate was used. D. Relative content of cofactors after 60 min, Ca (NCDH + NAD → NCD + NADH), 5 or 50 mM pyruvate was used. For the ME groups, 0.4 U/mL of ME was added, and for the ME∗ groups, 0.4 U/mL of ME∗ was added. For the ME&ME∗ groups, both 0.2 U/mL of ME and 0.2 U/mL of ME∗ were added. NO ENZYME groups were performed in the absence of malic enzymes. All reactions were incubated with 2 mM oxidative cofactor, 2 mM reductive cofactor, 100 mM NaHCO_3_, and 2 mM MgCl_2_ in 100 mM HEPES (pH 7.5) at 37 °C. 5 mM pyr and 50 mM pyr represent the initial concentrations of pyruvate used in the reaction was 5 mM and 50 mM, respectively. Experiments were done in triplicate, and error bars indicate standard deviation.

The reversible interconversion between pyruvate and malate serves as a bridge linking transhydrogenation. Consequently, the initial concentration of pyruvate influences the rate of hydrogen transfer. As anticipated, after 120 min of reaction with 0.5 mM pyruvate, the hydride conversion of the ME&ME∗ group was 21.8 %. As the pyruvate concentration gradually increased, under the same conditions, the hydride conversion of the ME&ME∗ group reached 72.5 % when 100 mM pyruvate was used. The conversion increased with the concentration of pyruvate, as shown in Fig. 3A, which indicated that pyruvate enhanced the efficiency of the transhydrogenation.

Subsequently, we examined the variation of hydride conversion efficiency over time. Referencing the concentrations of pyruvate in typical *E. coli* and yeast cells, which were estimated around 4 mM and 9 mM, respectively [27], as well as the fact that pyruvate can reach up to 50 mM or higher in engineered strains, we separately assessed the efficiency of hydrogen transfer under conditions of 5 mM and 50 mM pyruvate. At a pyruvate concentration of 50 mM, the transhydrogenation (Ac, NADH + NCD → NAD + NCDH) reached nearly maximal conversion of 66 % after 60 min, as demonstrated in Fig. 3B. However, when the pyruvate concentration was 5 mM, the hydride conversion continued to increase over a 2-h period, reaching a conversion of 53.8 % at 120 min. This observation further supports the notion that higher concentrations of pyruvate accelerate the reaction towards equilibrium. All of above indicated that the reaction proceeded efficiently, with the system achieving a steady state in a relatively short time frame, making it suitable for use in dynamic metabolic processes.

To further verify that the cooperation of two malic enzymes is necessary for electron transfer between natural and non-natural redox cofactors, we evaluated the hydrogen transfer efficiency with only one of malic enzymes present. As shown in Fig. 3C and D, the ME&ME∗ group containing both ME and ME∗ exhibited the highest hydrogen transfer efficiency after 60 min. In the Ac reaction (NADH + NCD → NAD + NCDH), the ME&ME∗ group demonstrated that 43.2 % and 57.6 % of NCD were reduced to NCDH at 5 mM and 50 mM pyruvate, respectively. It was much higher than the ME or ME∗ groups (each containing a single malic enzyme), where the reduction of NCD to NCDH remained below 15 %. A similar trend was observed in the Ca reaction (NCDH + NAD → NCD + NADH): 48.0 % (5 mM pyruvate) and 54.7 % (50 mM pyruvate) of NAD was reduced to NADH in the ME&ME∗ group, while the ME or ME∗ groups showed only about 10 %–15 % reduction of NAD to NADH. Meanwhile, we observed that in the ME∗ group of Ca reaction (NCDH + NAD → NCD + NADH), 15.0 % (5 mM pyruvate) and 37.5 % (50 mM pyruvate) of NCDH were oxidized to NCD, but only about 9.2 % and 13.1 % of NAD were reduced to NADH, respectively. In addition to the spontaneous oxidation of NCDH, a critical issue might be the trapping of reducing power in malate due to the absence of an enzyme that utilizes NAD, resulting in incomplete transfer of reducing power from NCDH to NADH. A similar issue might exist in the ME group of Ac reactions. However, since ME had noticeable capacity for NCD utilization, the retention of reducing power was less pronounced. In the ME&ME∗ group of the Ca reaction, the presence of enzymes that utilize both NAD(H) and NCD(H) reduced the retention of reducing power among intermediate products. In this group, 47.1 % (5 mM pyruvate) and 59.3 % (50 mM pyruvate) of NCDH were oxidized to NCD, with 48.0 % and 54.7 % of NAD reduced to NADH. These results confirmed that the combination of both malic enzymes is crucial for overall efficiency of the transhydrogenation reaction. In contrast, when only one type of malic enzyme—either ME or ME∗—was present, the transhydrogenation efficiency was considerably lower, even if the same total enzyme activity units were added. Taken together, our observations highlighted the synergistic nature of combining different malic enzymes for a high-yield cofactor-specific transhydrogenation.

These findings indicated that the ME-mediated transhydrogenation can efficiently promote hydrogen transfer between natural or non-natural nicotinamide cofactors. Furthermore, the intermediate substrates and cooperation between malic enzyme components are essential for efficient transhydrogenation. By altering the combination of enzymes, the varieties of cofactors regeneration can be further expanded. Regulating the energy flow between these cofactors opens up new avenues for optimizing metabolic fluxes, especially in the production of valuable compounds through engineered microorganisms in synthetic biology.

### 3.3 Biosynthesis of lactate regulated by the ME-mediated transhydrogenation *in vivo*

**Fig. 4:**
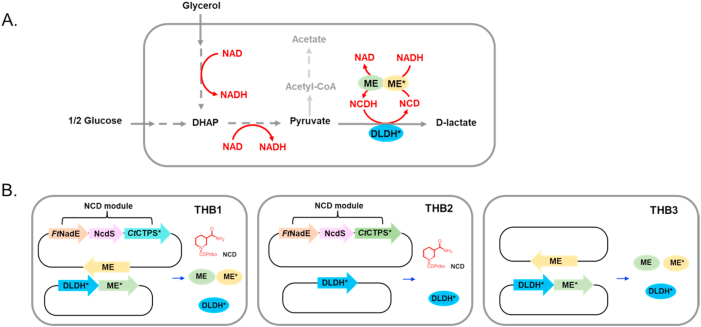
Biosynthesis of lactate mediated by the ME-mediated transhydrogenation *in E. coli*. A. Schematic diagram of ME-mediated transhydrogenation applied in lactate production. B. Schematic diagram of engineered strains for lactate production.

d-lactate, a versatile industrial chemical monomer, is produced by microorganisms through the catalytic reduction of pyruvate by d-lactate dehydrogenase (DLDH), typically utilizing nicotinamide cofactors as reducing agents in this process. Liu et al. engineered an NCD-preferring DLDH∗ (V152R/N213E mutant from *Lactobacillus helveticus)* [10]. We analyzed the kinetics of DLDH∗ toward NADH, NCDH, and NADPH, as shown in Table S3. The results demonstrated that its catalytic efficiency value (*k*_cat_/*K*_m_) for NCDH was approximately 9-fold and 255-fold higher than that for NADH and NADPH, respectively. The *K*_m_ value for NCDH was only 0.0711 mM, whereas those for NADH and NADPH were impractically high at 4.84 mM and 2.11 mM, respectively. These data indicated that DLDH∗ prefers NCDH over NADH or NADPH to catalyze lactate synthesis *in vivo*. Based on this, the ME-mediated transhydrogenation has the potential to regulate the d-lactate production driven by NCDH in cells (Fig. 4A).

To validate the regulation of lactate biosynthesis by the ME-mediated transhydrogenation *in vivo*, four *E. coli* strains THB1, THB2, THB3 and THB4 were constructed, as shown in Table S1. To avoid interference from lactate synthesized by the strain itself in experimental analysis, BW25113(*△ldhA*, *dld::cat*) [28] with endogenous lactate dehydrogenase knocked out was used as the host for lactate biosynthesis. The strain THB1 contains NCD synthesis module, ME-mediated transhydrogenation module (ME and ME∗), and lactate production module (DLDH∗). To balance the activities of ME, ME∗, and DLDH∗, ME was cloned onto a low-copy plasmid, whereas ME∗ and DLDH∗ which have relatively low activity compared to ME, have been cloned onto a plasmid with high copy number to achieve higher levels of protein expression (Fig. 4B). Compared to THB1, strain THB2 lacks their corresponding genes for ME and ME∗, whereas THB3 and THB4 lacks genes responsible for the NCD module and DLDH∗, respectively. Resting *E. coli* cells were used to evaluate the effectiveness of the ME-mediated transhydrogenation *in vivo.* Notably, THB4 exhibited undetectable d-lactate production under the same conditions (with glucose feeding and glycerol feeding), confirming that d-lactate synthesis was mediated by DLDH∗. Other results from THB4 were not displayed or analyzed further.

**Fig. 5:**
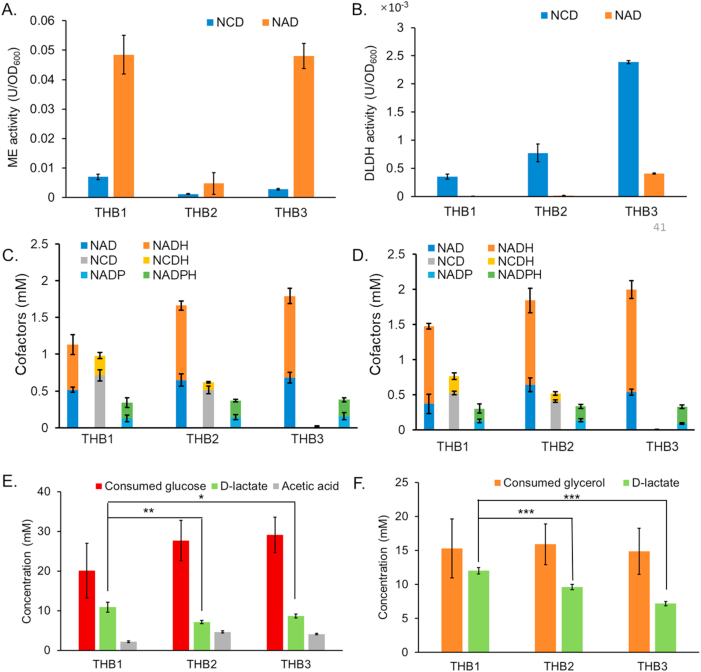
Biosynthesis of lactate mediated by the ME-mediated transhydrogenation using glucose and glycerol as substrates. A. Total malic enzyme activity of crude cell extracts of the engineered strains with NCD (blue bar) and NAD (orange bar). B. d-lactate dehydrogenase activity of crude cell extracts of the engineered strains with NCD (blue bar) and NAD (orange bar). C. Intracellular cofactor concentrations in engineered strains at 30-min resting cell catalysis under glucose conditions. D. Intracellular cofactor concentrations in engineered strains at 30-min resting cell catalysis under glycerol conditions. E. Glucose consumption and organic acid production by resting cells after 4 h when glucose was used. F. Glycerol consumption and d-lactate production by resting cells after 4 h when glycerol was used. For resting cells catalysis, about 2 × 10^10^ cells were incubated in HEPES (pH 7.5) in the presence of 100 mM glucose or glycerol at 37 °C with shaking at 200 rpm. All data represented the mean of three biologically independent samples and error bars indicated standard deviation. Group mean differences were evaluated by one-way ANOVA followed by Dunnett's post-hoc test, ∗p < 0.05, ∗∗p < 0.01 and ∗∗∗p < 0.001.

We detected the NCD- and NAD-linked activities of both malic enzyme (ME) and d-lactate dehydrogenase (DLDH) in the crude extracts of the engineered strains. THB1 and THB3 exhibited ME activity as expected. In contrast, THB2, which lacked expression of additional ME or ME∗, displayed minimal ME activity (Fig. 5A). Furthermore, it was found that THB1, THB2 and THB3 all exhibited DLDH activity towards NCD, with specific activities of 0.35 × 10^−3^ U/OD_600_, 0.77 × 10^−3^ U/OD_600_ and 2.39 × 10^−3^ U/OD_600_, respectively (Fig. 5B). DLDH∗ showed a strong preference for NCD in the cell, enabling it to effectively utilize NCDH regenerated by the ME-mediated transhydrogenation for lactate production. Notably, the activity of DLDH∗ in THB1 was relatively low, presumably due to resource limitations arising from the overexpression of various other proteins in this strain.

As a next step, d-lactate production of engineered *E. coli* strains was evaluated using resting cells. Experiments were conducted with 100 mM glucose or glycerol as the substrate at pH 7.5, 37 °C, and 200 rpm shaking. We evaluated the consumption of carbon sources (glucose or glycerol) and the production of d-lactate. Meanwhile, when glucose was used, we also assessed the level of acetic acid, an important byproduct generated during lactate synthesis from glucose by *E. coli*.

As an easily utilizable substrate, glucose can provide NADH to cells rapidly. We detected the levels of nicotamide cofactors in the engineered strains (Fig. 5C). It revealed that THB1 had 1.13 mM NAD(H) and 0.98 mM NCD(H), and THB2 had 1.66 mM NAD(H) and 0.62 mM NCD(H) at 30 min of resting cell biotransformation. Both THB1 and THB2 produced significant amounts of NCD(H) as expected. THB3 had 1.79 mM NAD(H), and no NCD(H) was detected as expected. NADP(H) levels were comparable across all engineered strains, with total concentrations of approximately 0.35 mM. In THB1, the NCDH concentration reached 0.26 mM with an NCDH/NCD ratio of 0.37, whereas THB2 exhibited only 0.10 mM NCDH and an NCDH/NCD ratio of 0.20. Given that DLDH exhibits a *K*_m_ of 0.07 mM for NCDH, the intracellular NCDH concentration could theoretically provide the driving force for lactate production. Further analysis of the NCDH/NCD ratio during resting cell catalysis revealed a higher proportion of NCDH in THB1 compared to THB2 (Fig. S3A), indicating that the ME-mediated transhydrogenation facilitated reducing power transfer to NCDH.

When glucose was used, at 4 h, THB1 consumed 20.1 mM glucose and accumulated 10.8 mM d-lactate, achieving a theoretical yield of 27.0 %. In comparison, THB2 consumed 27.7 mM glucose and accumulated 7.1 mM d-lactate, with a theoretical yield of 12.9 %. And THB3 consumed 29.1 mM glucose and accumulated 8.7 mM d-lactate, reaching a theoretical yield of 14.9 %. Meanwhile, the accumulation of acetic acid in the three engineered strains were 2.13 mM, 4.64 mM and 4.08 mM, respectively (Fig. 5E). Compared to THB2 and THB3, the d-lactate accumulation and yield of THB1 was significantly increased, while the byproduct acetic acid was reduced. This indicates that the ME-mediated transhydrogenation effectively directed carbon flux towards the NCD-mediated lactate pathway. Despite the absence of the ME-mediated transhydrogenation module or NCD synthesis module, THB2 and THB3 still had considerable d-lactate accumulation. This might be due mainly to the residual activity of DLDH∗ towards NADH, and to a lesser extent to interactions with other unknown metabolic processes involving the redox couple NCD/NCDH, as evidenced by the detection of NCDH in THB2.

In addition, we also use glycerol, which is surplus in biodiesel production, as the substrate for d-lactate production. Importantly, glycerol exhibits a thermodynamically favorable standard redox potential for reducing pyruvate [34,35]. Moreover, glycerol is a more efficient electron donor as it can be readily converted to DHAP for lactate production without dissipating reducing equivalents to other electron acceptors through multiple intermediate steps that are commonly required to metabolize other substrates like glucose. Two alternative routes have been reported for DHAP formation in *E. coli* [36]: the GlpK-GlpD/GlpABC route under respiratory conditions and the GldA-DhaKLM route under fermentative conditions. When glycerol is converted to pyruvate through the GldA-DhaKLM route, the NADH generated exceeds that required for the synthesis of d-lactate from pyruvate. This excess NADH can theoretically accumulate within the cell, driving the ME-mediated transhydrogenation to produce NCDH and ultimately promoting lactate synthesis. Similar to the cofactor profiles under glucose conditions, comparable NCD(H) levels were detected in both THB1 and THB2, with THB1 exhibiting a higher NCDH/NCD ratio (Fig. 5D and Fig. S3B). Under glycerol conditions, the NCDH of THB1 was 0.24 mM with an NCDH/NCD ratio of 0.45, whereas THB2 had only 0.13 mM and an NCDH/NCD ratio of 0.26 at 30 min of resting cell catalysis. Furthermore, our observations revealed that THB1 exhibited a notably higher lactate production compared to THB2 and THB3. As shown in Fig. 5F, at 4 h, THB1 consumed 15.3 mM glycerol and accumulated 12.0 mM d-lactate, achieving a theoretical yield of 78.6 %. In comparison, THB2 consumed 15.9 mM glycerol and accumulated 9.6 mM d-lactate, with a theoretical yield of 60.5 %. Meanwhile, THB3 consumed 14.9 mM glycerol and accumulated 7.2 mM d-lactate, reaching a theoretical yield of only 48.3 %.

Since previous tests showed no significant differences in cofactor levels between THB1 and THB2, and the activity of DLDH in THB2 and THB3 was much higher than THB1, we attribute the increased of d-lactate production and yield in THB1 in both substrates to the ME-mediated transhydrogenation. This system enabled the transhydrogenation between NADH and NCD *in vivo*, resulting in the formation of NCDH, which was utilized by DLDH∗ to enhance lactate production, meanwhile decreasing the byproduct acetic acid. This suggests that the ME-mediated transhydrogenation efficiently transfers reducing equivalents to the NCD-mediated lactate pathway, thereby promoting the production of d-lactate *in vivo*. The results demonstrate the critical role of the ME-mediated transhydrogenation in optimizing reducing equivalents utilization and directing metabolic flux toward lactate production. The synergy between non-natural cofactor-mediated bioorthogonal system and the ME-mediated transhydrogenation holds great promise for metabolic engineering applications targeting biochemicals.

We have demonstrated that elevated pyruvate concentrations could enhance the efficiency of ME-mediated transhydrogenation *in vitro*; therefore, optimizing pyruvate level should augment the system's functionality *in vivo*. Alternatively, the efficiency may be enhanced by improving intracellular CO_2_ availability. CO_2_ is produced by *E. coli* through a variety of means, like TCA cycle, pyruvate-formate lyase, and the pentose phosphate pathways. Potential strategies for enhancing CO_2_ availability include optimizing the fermentation process of microbial strains or introducing carbonic anhydrase to catalyze the reversible conversion between HCO_3_^−^ and CO_2_ [37,38]. Furthermore, previous studies employed sacrificial regeneration systems (such as phosphite or formate) to drive energy for non-natural cofactors mediated pathways [11,39,40], however, the inherent metabolic burden imposed by these exogenous reducing agents may limit their practical applicability in cells. In contrast, the ME-mediated transhydrogenation offers advantages in directing reducing equivalents for specific utilization. These strategies may integrate to achieve complementary functionalities in the future, and such hybrid systems could potentially address the limitations of individual approaches, broadening the application possibilities of non-natural cofactors in synthetic biology.

## 4 Conclusions

This study demonstrates the potential of using ME-mediated transhydrogenation as a novel approach for precise regulation of redox cofactors in metabolic engineering. By facilitating the dynamic exchange of reducing equivalents between natural nicotinamide cofactors and the non-natural one NCD, this system enables directing the reducing power into redox reactions of interest, without a complete dependence on native cofactors, thereby preserving their role in essential biological processes. We tested three types of malic enzymes with distinct cofactor specificities, namely, wild-type ME, ME∗ and MaeB preferring NAD, NCD and NADP, respectively, to facilitate transhydrogenation, and demonstrated an efficient transfer of reducing equivalents between different malic enzyme pairs, highlighting the flexibility and precision of this system in regulating energy fluxes within cells.

*In vitro* experiments confirmed the successful hydrogen transfer between various nicotinamide cofactors, enabling efficient hydride conversion. Subsequently, we found the intermediate substrate pyruvate boosts transhydrogenation. More importantly, the synergy between specific malic enzymes dependent on distinct cofactors is essential to mediate transhydrogenation from natural to non-natural cofactors. When ME-based system was applied *in vivo*, we observed a significant increase in d-lactate production. When glucose was used, the d-lactate titer was 1.52 times higher than that of the control, with a 14.1 % increase in yield and a decrease in byproducts. Similarly, when glycerol was used, the d-lactate titer was 1.45 times that of the control, and the yield increased by 18.1 %. This demonstrates that the ME-mediated transhydrogenation effectively directs reducing equivalent towards target products, thereby improving metabolic precision and efficiency.

Overall, this work presents ME-mediated transhydrogenation as a powerful tool for optimizing metabolic processes. Besides the conversion of NADH to NCDH to drive lactate production in this study, the approach may be applied to dynamically regulate the balance between other natural or non-natural redox cofactors, thus averting adverse impacts on growth and metabolism caused by inappropriate distribution of reducing power. The ability to manipulate natural and non-natural cofactors opens new possibilities for enhancing the production of high-value biochemicals and optimizing redox biocatalysis in synthetic biology and engineered microbial systems.

## CRediT authorship contribution statement

**Haizhao Xue:** Writing – original draft, Visualization, Methodology, Investigation, Formal analysis, Data curation, Conceptualization. **Yinghan Hu:** Writing – review & editing, Resources, Formal analysis. **Aabid Manzoor Shah:** Writing – review & editing. **Xueying Wang:** Resources, Funding acquisition. **Xiaojia Guo:** Resources. **Yanzhe Huang:** Writing – review & editing, Resources. **Zongbao K. Zhao:** Writing – review & editing, Conceptualization.

## **Declaration of competing interest**

The authors declare that they have no known competing financial interests or personal relationships that could have appeared to influence the work reported in this paper.
