# Supplementary material for: Malic enzyme-based system for transhydrogenation between nicotinamide cofactors
Source: Synth Syst Biotechnol. 2025 Aug 21;11:10–8. doi: 10.1016/j.synbio.2025.08.009 (PMC12492669; doi:10.1016/j.synbio.2025.08.009)
Supplement: Multimedia component 1 [file mmc1.docx]

**Supplementary** **Material**

**Malic enzyme-based system for transhydrogenation between nicotinamide cofactors**

**Contents**

**1. Tables**

Table S1. Strains used in this study

Table S2. Primers used in this study

Table S3. Kinetic parameters of ME, ME* and DLDH* for cofactors

**2. Figure**

Fig. S1 Schematics of enzymatic cycling assays for NAD(H) and NCD(H)

Fig. S2 Expression and purification of three malic enzymes

Fig. S3 Ratio of NCDH/NCD in engineered strains during resting cell catalysis for lactate production

Fig. S4 Kinetic profiles of ME, ME* and DLDH* with cofactors

**3. References**

**Table S1.** Strains used in this study.

| Plasmids and strains | Genotype or characteristic | Resource |
| --- | --- | --- |
| Plasmids |  |  |
| pET24b | Blank plasmid for protein expression | Novagen |
| pET24b-ME | pET24b with ME | [1] |
| pET24b-ME* | pET24b with ME mutant ME*(L310R/Q401C) | [2] |
| pUC-P15A-Para-FtNadE-c-his-NcdS-2-CtCTPS* | Expression for NCD module with P15A ori | [3] |
| p15A-NCD-ME | pUC-P15A-Para-FtNadE-c-his-NcdS-2-CtCTPS*, ME expressed with lac promoter | This study |
| pK-DLDH* | pK, DLDH mutant DLDH*(V152R/N213E) expressed with lac promoter | Our lab |
| pK-ME* | pK, ME* expressed with lac promoter | Our lab |
| pK-ME*-DLDH* | pK, ME*-rbs-DLDH* expressed with lac promoter | Our lab |
| P15A-ME | pUC-P15A with ME expressed | This study |
| Strains |  |  |
| *E. coli* BL21 (DE3) | F-, *dcm, ompT, hsdS (rB-, mB-), gal, λ*(DE3) | Novagen |
| BL21(DE3) (ME*) | BL21(DE3); pET24b-ME* | [2] |
| BL21(DE3) (ME) | BL21(DE3); pET24b-ME | [1] |
| *E. coli* K-12 (MaeB) | *E. coli* K-12; pCA24N-MaeB | Our lab |
| BW25113 (*△ldhA, dld::cat*) | rrnB3, *ΔlacZ4787, hsdR514, Δ(araBAD)567, Δ(rhaBAD)568 rph-1, △ ldhA,dld::cat* | [3] |
| THB1 | BW25113 (*△ldhA, dld::cat*); p15A-NCD-ME, pK-ME*-DLDH* | This study |
| THB2 | BW25113 (*△ldhA, dld::cat*); pUC-P15A-Para-FtNadE-c-his-NcdS-2-CtCTPS*, pK-DLDH* | This study |
| THB3 | \| BW25113 (*△ldhA*, *dld::cat*); P15A-ME, pK-ME*-DLDH* \| This study \| \| --- \| --- \| | This study |
| THB4 | BW25113 (*△ldhA, dld::cat*); P15A-NCD-ME, pK-ME* | This study |

**Table S2.** Primers used in this study.

| Primers | Sequence (5’ to 3’) |
| --- | --- |
| 24010502F | GATTATTGGCTGCTTGATGGGCGACTGGATTTCCGGATATAGTTCCTCCTTTCAG |
| 24010502R | GCTTCCGGCTCGTATGTTGTGTGGAATTGTGAGCGGATAACAATTCCCCTCTAGAAAT |
| 24010501pR | AAATCCAGTCGCCCATCAAG |
| 24010501pF | CTCACAATTCCACACAACATACG |

**Table S3.** **Kinetic parameters of ME, ME* and DLDH* for cofactors**

| Enzyme | Reaction process | Cofactor | *k*_cat_ | *K*_m_ | *k*_cat_/*K*_m_ | Source |
| --- | --- | --- | --- | --- | --- | --- |
|  |  |  | [s^-1^] | [mM] | [mM^-1^ s^-1^] |  |
| ME | Oxidative decarboxylation | NAD | 57.3 ±0.4 | 0.27 ± 0.01 | 214.8 | [2] |
|  |  | NCD | 26.1 ± 4.1 | 9.4 ± 1.9 | 2.8 |  |
|  |  | NADP ^a^ | 0.58 ± 0.05 | 11.5 ± 1.2 | 0.05 | This study |
|  | Reductive carboxylation | NADH ^b^ | 1.88 ± 0.35 | 1.08 ± 0.35 | 1.74 |  |
|  |  | NCDH ^b^ | n.d. | | |  |
|  |  | NADPH ^b^ | 0.01 ± 0.00 | 0.52 ± 0.12 | 0.02 |  |
| ME* | Oxidative decarboxylation | NAD | 10.4 ± 1.2 | 3.8 ± 0.4 | 0.36 | [2] |
|  |  | NCD | 158.2 ± 17.8 | 1.02 ± 0.17 | 154.6 |  |
|  |  | NADP ^a^ | 0.01 ± 0.00 | 2.86 ± 0.44 | 0.00 | This study |
|  | Reductive carboxylation | NADH ^b^ | 0.03 ± 0.00 | 1.15 ± 0.11 | 0.02 |  |
|  |  | NCDH ^c^ | 1.17 ± 0.15 | 1.89 ± 0.36 | 0.62 |  |
|  |  | NADPH ^c^ | n.d. | | |  |
| DLDH* | Lactate oxidation | NAD | 0.08 | 1.60 ± 0.22 | 0.05 | [4] |
|  |  | NCD | 2.95 ± 0.27 | 1.38 ± 0.34 | 2.1 |  |
|  |  | NADP ^a^ | n.d. | | | This study |
|  | Pyruvate reduction | NADH ^b^ | 265 ± 58 | 4.84 ± 1.47 | 54.7 |  |
|  |  | NCDH ^c^ | 33.6 ± 1.4 | 0.07 ± 0.01 | 472 |  |
|  |  | NADPH ^c^ | 3.91 ± 0.88 | 2.11 ± 0.68 | 1.85 |  |

Note: n.d. denotes that enzymatic activity was not detectable within the tested range of cofactor concentrations. ^a^ NADP concentration ranged from 20 to 5000 μM. ^b^ NADH concentration ranged from 20 to 2500 μM. ^c^ NCDH and NADPH concentrations ranged from 20 to 1250 μM. Assays were done in triplicate, and the data represent the average ± standard deviation. Kinetic profiles were also shown in Figure S4.

**Fig. S1 Schematics of enzymatic cycling assays for NAD(H) and NCD(H)**


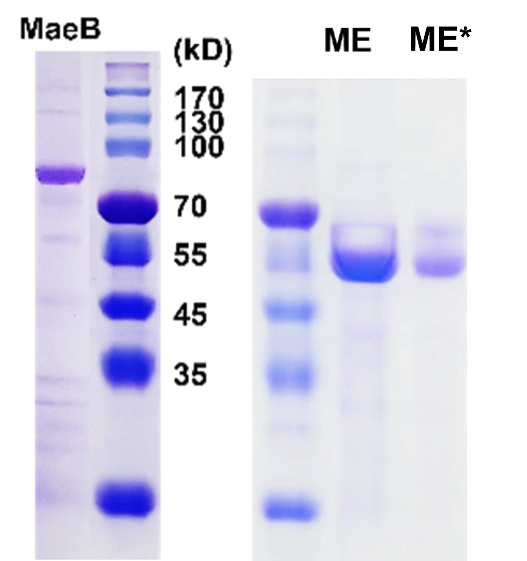


**Fig. S2 Expression and purification of three malic enzymes**


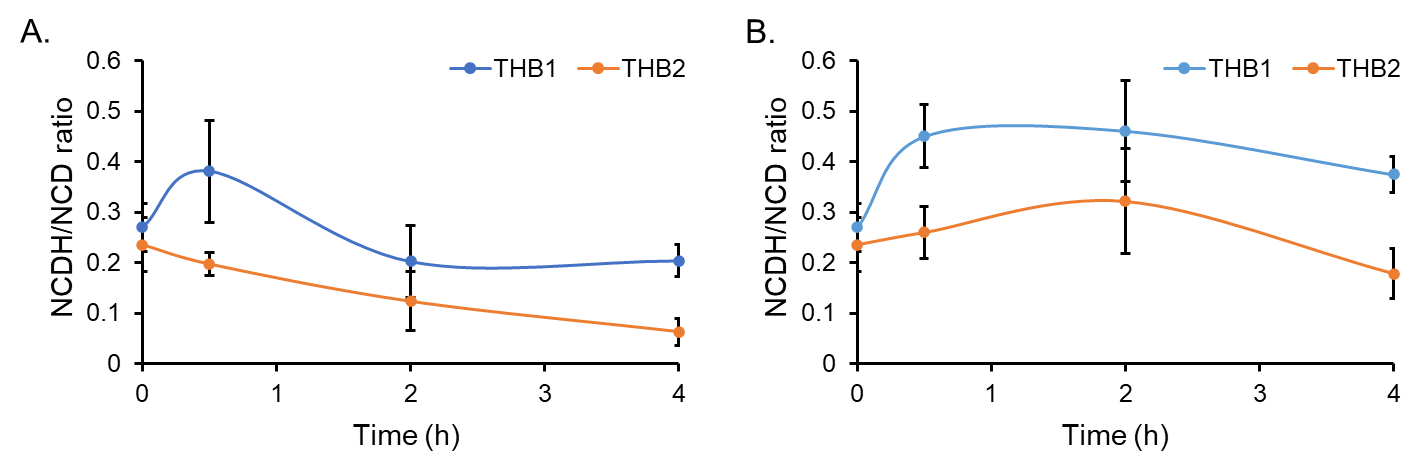


**Fig. S3 Ratio of NCDH/NCD in engineered strains during resting cell catalysis for lactate production.** A. Glucose as carbon source; B. Glycerol as carbon source. Experiments were done in triplicate and error bars indicate s.d.

**Fig. S4 Kinetic profiles of ME, ME* and DLDH* with cofactors.** Data were fitted with the Michaelis−Menten equation. Experiments were done in triplicate and error bars indicate s.d.

1. Wang J, Tan H, Zhao ZK. Over-expression, purification, and characterization of recombinant NAD-malic enzyme from *Escherichia coli* K12. Protein Expr Purif. 2007;53:97–103. https://doi.org/10.1016/j.pep.2006.11.017
2. Ji D, Wang L, Hou S, Liu W, Wang J, Wang Q, et al. Creation of bioorthogonal redox systems depending on nicotinamide flucytosine dinucleotide. J Am Chem Soc. 2011;133:20857–62. https://doi.org/10.1021/ja2074032
3. Wang X, Feng Y, Guo X, Wang Q, Ning S, Li Q, et al. Creating enzymes and self-sufficient cells for biosynthesis of the non-natural cofactor nicotinamide cytosine dinucleotide. Nat Commun. 2021;12:2116. https://doi.org/10.1038/s41467-021-22357-z
4. Liu Y, Li Q, Wang L, Guo X, Wang J, Wang Q, et al. Engineering *d*‐lactate dehydrogenase to favor a non‐natural cofactor nicotinamide cytosine dinucleotide. ChemBioChem. 2020;21:1972–5. https://doi.org/10.1002/cbic.201900766
